# The Effects of Cold Saponification on the Unsaponified Fatty Acid Composition and Sensory Perception of Commercial Natural Herbal Soaps

**DOI:** 10.3390/molecules23092356

**Published:** 2018-09-14

**Authors:** Natalia Prieto Vidal, Oludoyin Adeseun Adigun, Thu Huong Pham, Abira Mumtaz, Charles Manful, Grace Callahan, Peter Stewart, Dwayne Keough, Raymond Horatio Thomas

**Affiliations:** School of Science and the Environment/Boreal Ecosystem Research Facility, Grenfell Campus, Memorial University of Newfoundland, 20 University Drive, Corner Brook, NL A2H 5G4, Canada; doyenhoney@yahoo.com (O.A.A.); tpham@grenfell.mun.ca (T.H.P.); abiramumtaz@gmail.com (A.M.); cmanful@gmail.com (C.M.); grace.callahan99@gmail.com (G.C.); pstewart@grenfell.mun.ca (P.S.); Dwayne.Keough@algomau.ca (D.K.)

**Keywords:** natural herbal soap, fatty acid composition, unsaponified fatty acids, sensory perception

## Abstract

Saponification is the process in which triglycerides are combined with a strong base to form fatty acid metal salts during the soap-making process. The distribution of unsaturated and saturated fatty acid determines the hardness, aroma, cleansing, lather, and moisturizing abilities of soaps. Plant extracts, such as rosemary, vegetable, and essential oils are frequently added to soaps to enhance quality and sensory appeal. Three natural soaps were formulated using cold saponification to produce a base or control bar (BB), hibiscus rosehip bar (H), and a forest grove bar (FG). Rosemary extract (R) or essential oil (A) blends were added as additives to each formulation prior to curing to evaluate the effects of natural plant additives on the lipid composition and sensory characteristics of these natural herbal soaps. A total of seven natural soaps, three without additives (BB, H, FG) and four with additives (BBR, HA, FGR, FGA), were manufactured and studied. The majority (86–99%) of the polyunsaturated fatty acids (5.0–7.0 µg/mg) remained unsaponified in the manufactured natural soaps regardless of feedstock used. Principal component analysis (PCA) analyses showed the unsaponifiable fatty acids were different in the hibiscus bar compared to the other bars. There was a very strong correlation between the content of unsaponified C18:3n3 and C18:1n9 in all natural soaps. These results indicate that unsaponified fatty acids are important contributors to the quality and overall sensory perception and preference of natural herbal soaps following manufacturing by cold saponification.

## 1. Introduction

The global bar soap industry is worth approximately US$186 billion. Current trends in consumer preference have shown an increase in demand for the use of natural ingredients in personal skin care and cosmetics products. This has resulted in a steady increase in small- and medium-sized artisan hand-made or homemade soap businesses offering a variety of products with a range of natural ingredients to supply this niche market. Formulation of specialty hand-made natural soap bars by artisan soap makers involves a skillful combination of the ingredients, thoughts, and artistic creativity to produce high-quality soap bars with superior sensory characteristics that resonates with consumers [1]. These sensory characteristics include fragrance, color, lather ability, moisturizing capabilities, hardness, skin compatibility, and chemical stability during storage and use [1,2,3,4]. Of these, aroma (fragrance) and moisturizing capabilities are considered the most influential determinants of consumer preference for natural soap products. Natural soaps are generally defined as alkali salts of fatty acids derived primarily from vegetable or plant oils used as soap feedstock, and contained natural fragrances and/or organic ingredients included as additives. Commercially, natural soaps are manufactured via either a cold or hot saponification process, where triglycerides in fats, oils, and/or free fatty acids used as feedstock are converted in the presence of a base (typically sodium or potassium hydroxide) to form fatty acid salts (soaps), glycerol, and free fatty acids [1,3]. Many artisanal soap makers prefer the cold saponification process due to the enhanced aesthetics of the finished product, potential superiority in retaining antioxidants, or the fragrance from essential oils, and creative flexibilities to customize each ingredient including the addition of fresh ingredients, such as fruits and vegetable purees, to obtain a desired end product. Cold saponification uses the heat generated from the combination of the fatty acids (acid) in the melted oils and fats with sodium hydroxide (base) to facilitate the saponification process, which takes 18–24 h to complete, and a further 3–4 weeks to cure the finished soaps [3]. The sensory and chemical characteristics of natural soaps are dependent on the manufacturing process, and the chemical composition of the feedstock materials used during formulation. For example, the type and purity of base (alkali) used determines the hardness and solubility of the finished soap. Sodium hydroxide produces harder, more durable soaps, while potassium hydroxide is used to produce soft soap bars or liquid soaps [2,5]. Typically, 70–85% of the total contents of natural herbal soap bars are composed of sodium salts of fatty acids (saponified fatty acids) derived from triglycerides or hydrolyzed fatty acids present in the soap feedstock [3]. These fatty acids play key roles in the performance of the natural soap, consumer preferences, and the cost of the finished products. Final performance is determined by the carbon chain length, degree of unsaturation (number of double bonds), and distribution and composition of the saponified fatty acids (alkali salts). Saturated fatty acids give light open foams (lather) and a solid, hard consistency, while unsaturated fatty acids provide moisturizing, conditioning, or skin nourishing properties [1,2,3,5,6]. 

The most commonly used oil sources in natural herbal soaps are mixtures of palm, coconut, olive, rice bran, and sunflower seed oils [1,3]. In some instances, animal fat may be blended with vegetable oils in the final formulation to modulate the soap performance. Vegetable oils tend to be associated with higher quality soaps [1,3,4]. As such, the use of animal fats is typically replaced with palm or coconut oils to enhance the quality and performance of natural soaps. Vegetable oils tend to be richer in polyunsaturated (C16:3, C18:2, and C18:3) fatty acids, while beef fat (tallow) tends to contain higher levels of long chain (C16:0–C18:0) saturated fatty acids [4,6,7]. Palm and coconut oils, on the other hand contain shorter chain length (C8:0–C14:0) saturated fatty acids. The shorter chain saturated fatty acids in coconut or palm oil increase the lathering profile of the final soap products due to enhanced solubility in water. However, fatty acids with 10 or fewer carbons are less desired because they can confer objectionable odors and irritate the skin. Conversely, longer (C16:0–C18:0) chain length fatty acids enhance the cleansing property of the soap, provide a longer lasting soap, and are devoid of objectionable odors [1,3,4]. However, the lathering ability is reduced due to decrease solubility in water with the increase in chain length [4]. Vegetable oils, such as soybean or olive oils, also contain significant levels of long chain saturated fatty acids (C16:0–C18:0), as well as high levels of mono (C18:1) and polyunsaturated (C18:2, C18:3, and C16:3) fatty acids. The solubility and moisturizing capabilities of natural herbals soaps will increase with the degree of unsaturated fatty acids present in the vegetable oils used as soap feedstock [1,5,7]. However, the double bonds found in unsaturated fatty acids are very susceptible to oxidation, which can occur during saponification, curing, and storage. Oxidation of the double bonds can produce shorter chain fatty acids, aldehydes, ketones, undesirable odors, and discoloration in the soaps, which affects the quality, sensory perception, and shelf life of the final product [1,3]. Natural antioxidants in the form of plant extracts are typically added as additives (1–8% of final soap composition) to suppress the oxidation of polyunsaturated fatty acids in natural herbal soaps. These include grape seeds or rosemary extracts, fruits, and vegetable purees. In addition to providing a source of natural antioxidants, these plant extracts are also used as colorants and to add fragrance. A key aesthetic for consumer acceptance of personal cleansing products is fragrance [3]. As such, artisanal natural soap manufacturers use a variety of exotic plant-based essential oils as additives to provide added consumer benefits, enhance the sensory perception, as well as modify the performance of their products [3]. Like the unsaturated fatty acids in the soap, essential oils are also susceptible to oxidation during saponification, storage, or curing. As a result, essential oils used as additives can modulate the odor profile and quality of the product during its lifetime if not properly formulated. Free fatty acids are another important subclass of additives used during the formulation and manufacturing of natural herbal soaps [2]. The free fatty acids can exist as unsaponified fatty acids in the final product following saponification and curing. Though the free fatty acids added as additives can decrease the odor and color stability of the final products, they are also known to play an important role in determining soap quality [2,3]. For example, free fatty acids can enhance the fragrance, moisturizing capabilities, foaming, or lather quality. These are important soap quality indices that can influence consumer preference and acceptance [2]. Thus, the free fatty acid content, though a minor component of the overall soap composition, can have significant influences on the overall quality and sensory characteristics, and in turn, consumer preference or acceptance of natural soaps [2,3]. The right blends of natural fats, oils, and additives (natural plant extracts, free fatty acids, essential oils, and antioxidants) are important factors to consider in the design and manufacture of natural soaps with the ideal physicochemical characteristics and sensory appeal to meet consumer’s demands [1,2,3]. Many artesian or hand-made soap makers use a combination of cold saponification, essential oils, and plant extracts as additives to manufacture various natural soaps. However, very little is known how the combination of cold saponification and the use of essential oils and plant extracts as additives: (1) how modulating the levels of unsaponified (or free) fatty acids in commercial natural soaps, and (2) how the composition of the unsaponified fatty acids in the manufactured soap influences the soap’s sensory quality and consumer’s perception of the finished products. This is of relevance considering the current global trends show an increase in the number of hand-made specialty natural soap businesses. Many of these businesses are home-based and use an array of additives and essential oils to manufacture their product, but have very little scientific information on how these ingredients can affect the unsaponified fatty acid content, quality, and consumer’s preference or acceptance of their products. We hypothesized that the composition of the feedstock and additives used to manufacture natural herbal soaps following cold saponification will significantly affect the level and composition of free or unsaponified fatty acids in the finished product, and that the unsaponified fatty acids, though a minor component of the soap composition, will have a major influence on the quality, sensory perception, and consumer preference of the final product. This study was designed to test this hypothesis. 

## 2. Results and Discussion

### 2.1. Effects of Feedstock Sources on the Unsaponified Fatty Acid Composition of Natural Herbal Soaps

**Figure 1:**
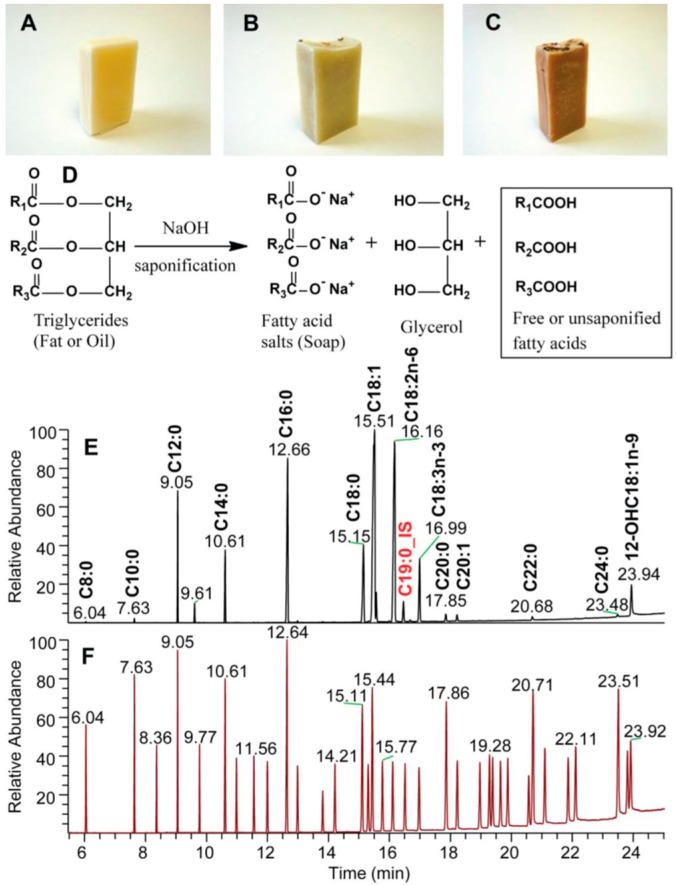
Base bar (**A**), forest grove (**B**), and hibiscus rose hip (**C**) natural soaps manufactured using cold saponification. (**D**) Chemical equation showing the cold saponification reaction associated with the manufactured natural soap products. Gas Chromatography/ Flame Ionization Detector (GC/FID) chromatogram of the fatty acid composition of a natural soap sample (**E**) relative to that of a commercial standard mix (**F**) of fatty acid standards (Supelco 37 components mix) used as reference during sample analyses.

**Figure 2:**
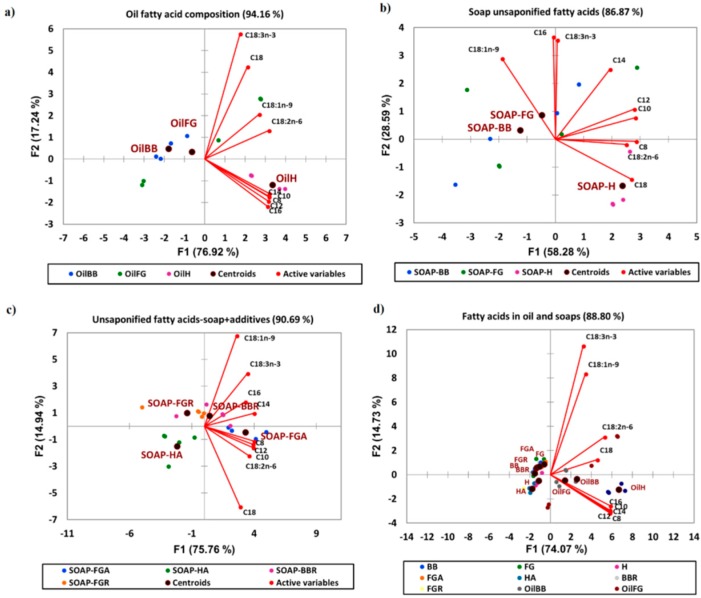
Principal component analysis (PCA) showing the similarities between different commercial soaps based on the fatty acids composition. Biplots showing clusters of (**a**) the oils used to make the natural soaps based on the fatty acid composition, (**b**) the three natural soaps made after cold saponification based on content of unsaponified fatty acids, (**c**) three natural soaps after additives were added based on unsaponified fatty acid composition, (**d**) oils and soaps with and without additives based on the fatty acid composition in oils (feedstock) and unsaponified fatty acids in soaps; *n* = 4 per experimental replicate. Unsaponified fatty acids are arranged based on the fatty acid composition with the number before the colon representing total number of carbons, while the numbers after the colon represents the total number and position of first double bonds (e.g., C16:3n3 = 16 carbons with 3 double bonds; first double located at carbon 3 counting from the terminal end. Natural soap acronyms: BB = base bar (control), FG = forest grove, H = Hibiscus rose hip. FGA = forest grove + essential oil and clay, HA = hibiscus + essential oil and clay, BBR = base bar + rosemary, FGR = Forest grove + rosemary, HR = hibiscus + rosemary.

Three natural soaps were manufactured in this study: BB, FG, and H (Figure 1). Vegetable oils are associated with the manufacturing of high quality natural soaps [2]. Consequently, a diverse mix of plants/vegetable oils were used as the feedstock ingredients in the production of all three natural soaps manufactured in this study. These included shea butter, coconut, palm, castor bean, olive, rice bran, and soybean oils. Interestingly, we observed that regardless of the source of plant or vegetable oil sources used as feedstock, the qualitative fatty acid composition was similar between all soap types (Table 1). However, significant quantitative differences were observed between saturated (28.17 ± 5.84 µg/mL vs. 70.36 ± 7.48 µg/mL) and polyunsaturated (5.36 ± 0.37 vs. 7.44 ± 0.23 µg/mL) fatty acids in the feedstock used to produce hibiscus rosehip soap (H) compared to the control (BB). As such, the Principal component analysis (PCA) analysis of the oils (Figure 2a) showed that the feedstock of both BB and FG were similar (clustered together) in composition, while the composition of the H feedstock was different (clustered in separate quadrants of the biplot) from that of the BB and FG. An increase in total saturated (C12:0 and C16:0) and polyunsaturated (C18:2n6) fatty acids were observed to be the major contributor to the quantitative differences in the fatty acids composition of the blended feedstock ingredients (Table 1). Free or unsaponified fatty acids are minor components (1–8%) of the overall composition of natural soaps produced following saponification of the fatty acids or triglycerides in the feedstock with an alkali base (sodium or potassium hydroxide). However, they can be major determinants of soap quality [2,3], having a significant influence on consumer’s preference or sensory perception of the finished products [2]. Many artisan soap makers use a variety of fats and oil sources as feedstock, as well as additives and essential oils to make natural soaps, but very little is known concerning how this affects the unsaponified fatty acid composition in their final product. The natural soaps manufactured in this study were superfatted (effective lye discount ratio) at 6%, and several sources of vegetable oils were used during cold saponification to manufacture the final products (Figure 1). Following cold saponification, we observed the presence of unsaponified fatty acids in all three natural soaps (BB, FG, and H), as anticipated. The content ranged from 8.90 ± 0.96 µg/mg of unsaponified saturated fatty acids in the control bar (BB) to 10.10 ± 0.73 µg/mg in the hibiscus bar (H) (Table 2). The following unsaponified saturated fatty acids were observed in all three natural soaps: C8:0, C10:0, C12:0, C14:0, C16:0, and C18:0, in accordance with findings reported previously in the literature [2]. Regardless of the sources of vegetable oils used as feedstock, the level of total unsaponified saturated fatty acids was not significantly different between the three kinds of natural soaps produced (Table 2). Conversely, the levels of total unsaponified monounsaturated fatty acids varied with the vegetable oil source used as feedstock. As such, a significantly lower level of C18:1n9 (oleic acid) was observed in the H soap bar (3.43 ± 0.36 µg/mg) compared to the control (4.57 ± 0.20 µg/mg) and FG (4.79 ± 0.95 µg/mg). Rice bran oil was added to the H soap bar, and the olive oil level reduced by 10% to accommodate the incorporation of the rice bran oil in the H bar formulation. This accounted for the reduced level of unsaponified C18:1n9 fatty acid observed in the H soap bar (Table 2) because oleic acid (C18:1n9) is the predominant fatty acid present in olive oil [7,8]. As such, the H soap was observed to have an unsaponified fatty acid composition distinct from that of the BB (control) and FG following principal component analyses (Figure 2b). Both the BB and FG clustered in the same quadrant of the biplot, and this grouping explained 86% of the total variance present in the samples based on the unsaponified fatty acid composition. Linoleic (C18:2n6) and linolenic (C18:3n3) acids were the unsaponified PUFAs observed in all three natural herbal soaps investigated in this study. The overall PUFA unsaponified fatty acid levels were similar in all three soap types (4.78 ± 0.05 to 5.40 ± 0.39 µg/mg), even though the levels of C18:3n3 was significantly lower in H (0.62 ± 0.04 µg/mg) compared to the BB (0.71 ± 0.05 µg/mg) and FG (0.70 ± 0.07 µg/mg) (Table 2). Ricinoleic acid (12-OH C18:1n9) obtained from castor beans was observed in the oil mix used as soap feedstock (Figure 1), but was absent as unsaponified fatty acid in the soaps following cold saponification. Collectively, these findings show that the source of vegetable oil used as feedstock can modulate the unsaponified fatty acid composition and levels in natural herbal soaps following cold saponification. The unsaponified monounsaturated fatty acids (C18:1n9) appeared to be the most responsive to the variations in the choice of vegetable oils used as soap feedstock and manufacturing via cold saponification. Rice bran oil appears to reduce the content of unsaponified mono-unsaturated fatty acids in natural herbal soaps, when incorporated in the formulation at the expense of olive oil. 

### 2.2. Effects of Natural Additives on the Unsaponified Fatty Acid Composition of Natural Herbal Soaps 

**Figure 3:**
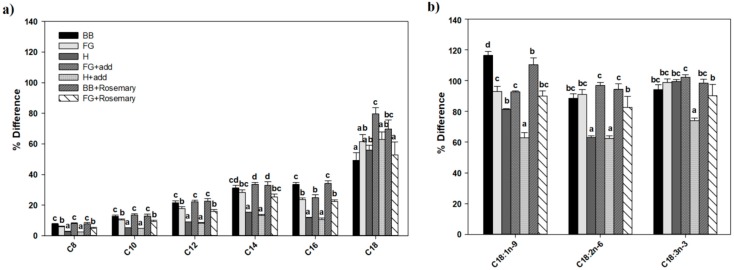
The effects of cold saponification and natural additives on the percent change in (**a**) saturated and (**b**) unsaturated unsaponified fatty acids in different commercial natural soaps. Values represent means ± standard errors. Means in the same row accompanied by different letters are significantly different between treatments at LSD = 0.05, *n* = 4 per experimental replicate.

Natural additives are commonly formulated into herbal soaps to modify the soap composition, quality, performance, and consumer’s sensory perception of the final product [3]. These include free fatty acids, essential oils, plant extracts, antioxidants, and colorants [1,2,3]. However, there is a paucity of information available to the large hand-made soap industry as to how this may affect the unsaponified fatty acid content in the soap produced. In the present study, essential oils + clay (A) or rosemary plant extracts (R) were added to the control (BB), FG, and H feedstocks. These samples are named BBR, FGR, FGA, and HA respectively. The BBA (base bar with additives) and HR (hibiscus with rosemary) samples were not included in this study because the BB, FG, and H serve as controls for the treatments with additives. We observed that incorporation of natural additives in the soap formulation modulated the unsaponified fatty acid composition present in the final products following manufacturing by cold saponification. BBR, FGR, FGA, and HA clustered in different quadrants of the biplot, indicating that inclusion of additives in BB, FG, and H modified the content of the unsaponified fatty acids in the formulated natural soaps. This clustering explained 90.69% of the total variance in the samples based on the unsaponified fatty acid content (Figure 2c). However, when the feedstock fatty acids (OilBB, OilFG, and OilH) and the unsaponified fatty acids of the manufactured soaps with (BBR, HA, FGR, and FGA) and without (BB, FG, and H) additives were included in the principal component analysis, the oils of the feedstock clustered together in quadrant 2, and FG and BB soaps with and without additives clustered together in quadrant 4, while H soaps with and without additives clustered together in quadrant 3 of the biplot (Figure 2d). These groups or clusters explained 88.8% of the total variances present in all the samples, and indicated that there are similarities in fatty acid composition of the oils used as feedstock, as well as the unsaponified fatty acids present in FG and BB or H, irrespective of whether additives were used. Both the samples with and without additives had the same unsaponified fatty acid composition, indicating the formulation of additives in the natural soaps had no qualitative effect on the unsaponified fatty acid composition in the final soap products (Table 2 and Figure 3). A surprising observation was that the overall total unsaponified saturated, MUFAs and PUFAs, were not altered by the inclusion of additives in the natural soap formulations (Table 2). One of the most striking findings observed in this study was that the majority (60–100%) of the unsaturated fatty acids (C18:1n9, C18:2n6, and C18:3n3) present in the feedstock used to manufacture all three natural soaps were present as unsaponified fatty acids in the final products following cold saponification (Figure 3). This observation was constant regardless of the vegetable or plant oil sources used as feedstock, and the inclusion of additives in the formulation. This is in contrast to the results reported by Bernecke and Maruska [2], who observed that the majority of unsaponified fatty acids present in the soap samples they evaluated were saturated. We note that we were unable to determine what saponification process or superfatting level (lye discount) was used to manufacture the soaps in the referenced study [2]. Also, in our study, we specifically focused on natural soaps with vegetable oils used as the source of the soap feedstock. These factors could account for the variation in quantitative levels of unsaponified fatty acids present in the natural herbal soaps evaluated in our study. We also observed that between 10% and 30% of the saturated fatty acids with chain lengths between C8:0 to C16:0 present in the natural soap feedstocks were retained as unsaponified fatty acids compared to between 40–80% for C18:0 saturated fatty acids. Though we observed that the exact quantity and quality of the unsaponified fatty acids present in the end products were not significantly affected by the additives used in the formulation following manufacturing by cold saponification (Table 2). We observed significant percent changes in the retention of unsaponified fatty acids in the final products when additives were used in the formulation (Figure 3). Essential oils and clay included as additives in the formulation reduced the percent retention of unsaponified C18:3n3 and C18:1n9 fatty acids in HA (Figure 3). Conversely, rosemary extract appears to enhance the retention of C18:0 unsaponified fatty acid in BBR (Figure 3). Taken all together, these findings suggest the use of additives as essential oils or plant extracts in natural soap formulation did not significantly alter the composition and levels of unsaponified fatty acids in the finished products following manufacturing by cold saponification. However, the additives significantly altered the percent change or retention of the feedstock fatty acids as unsaponified fatty acids in the finished products. The majority of the C18:0, C18:1n9, C18:2n6, and C18:3n3 fatty acids present in the feedstock remained as unsaponified fatty acids in the final products. That is, they were not converted to fatty acid salts (saponified) during the soap making process. In particular, the unsaturated fatty acids were not saponified when soaps were manufactured by cold saponification. This resulted in significant levels of unsaponified unsaturated fatty acids in the final products. This is of relevance because unsaponified fatty acids can modulate the quality and performance [2], as well as the shelf life of natural soaps. 

### 2.3. Phenolic, Antioxidant, and Oxidant Status in Natural Herbal Soaps 

**Figure 4:**
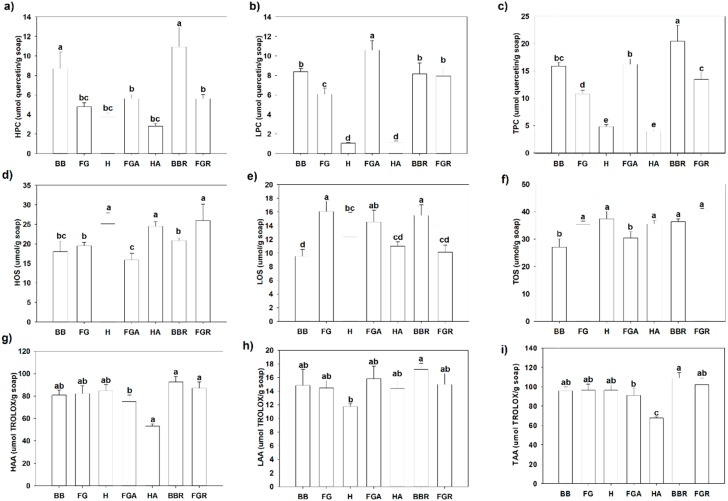
Total phenol, antioxidant, and oxidant content in natural soaps following cold saponification. (**a**,**d**,**g**) Hydrophilic, (**b**,**e**,**h**) lypophilic, and (**c**,**f**,**i**) total phenolic content, antioxidants activity, and oxidant content, respectively, in commercial soaps. Values represent means ± standard errors. Means in the same row accompanied by different letters are significantly different between treatments at LSD = 0.05, *n* = 4 per experimental replicate. Natural soap acronyms: BB = base bar (control), FG = forest grove, H = hibiscus rose hip, FGA = forest grove + essential oils and green clay, HA = hibiscus + essential oils and pink clay, BBR = base bar + rosemary, FGR = Forest grove + rosemary, HR = hibiscus + rosemary, HOS = hydrophilic oxidant status, LOS = lipophilic oxidant status, TOS = total oxidant status, HPC = hydrophilic phenolic content, LPC = lipophilic phenolic content, TPC = total phenolic content, HAA = hydrophilic antioxidant activity, LAA = lipophilic antioxidant activity, TAA = total antioxidant activity.

**Figure 5:**
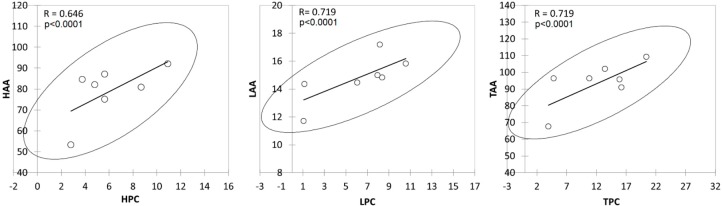
Scatter plots showing relationships between the phenolic content and antioxidant activities in different commercial natural soaps after cold saponification. HPC = hydrophilic phenolic content, LPC = lipophilic phenolic content, TPC = total phenolic content, HAA = hydrophilic antioxidant activity, LAA = lipophilic antioxidant activity, TAA = total antioxidant activity.

**Figure 6:**
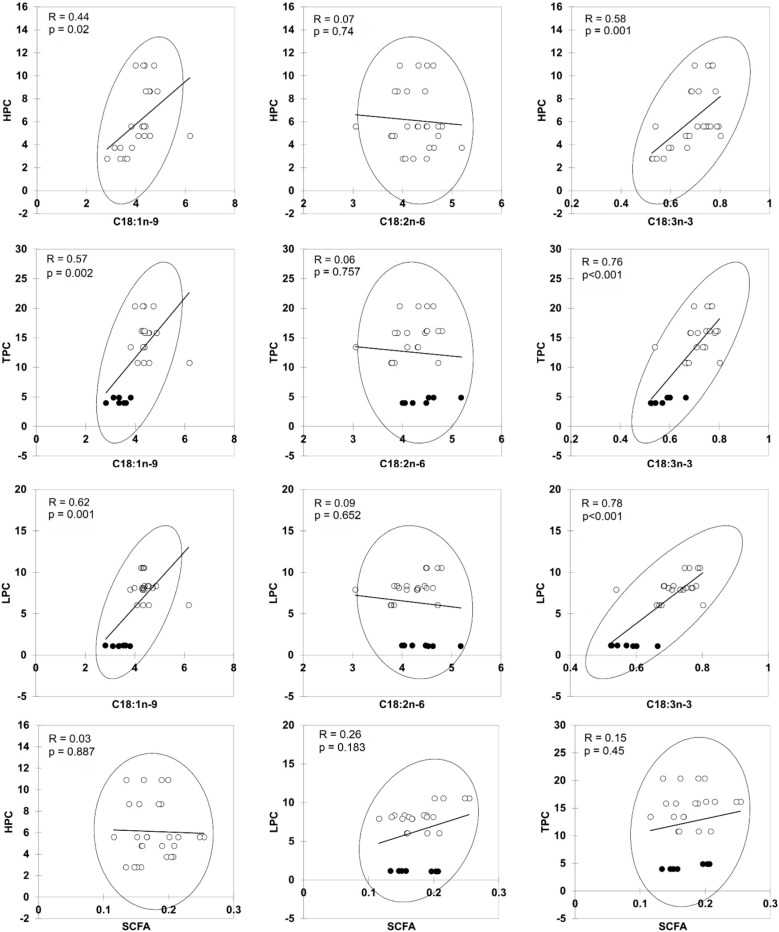
Scatter plots showing relationships between the phenolic content and unsaponified fatty acids in different commercial natural soaps after cold saponification. HPC = hydrophilic phenolic content, LPC = lipophilic phenolic content, TPC = total phenolic content. Short Chain Fatty acids (SCFA) = C8:0–C10:0.

**Figure 7:**
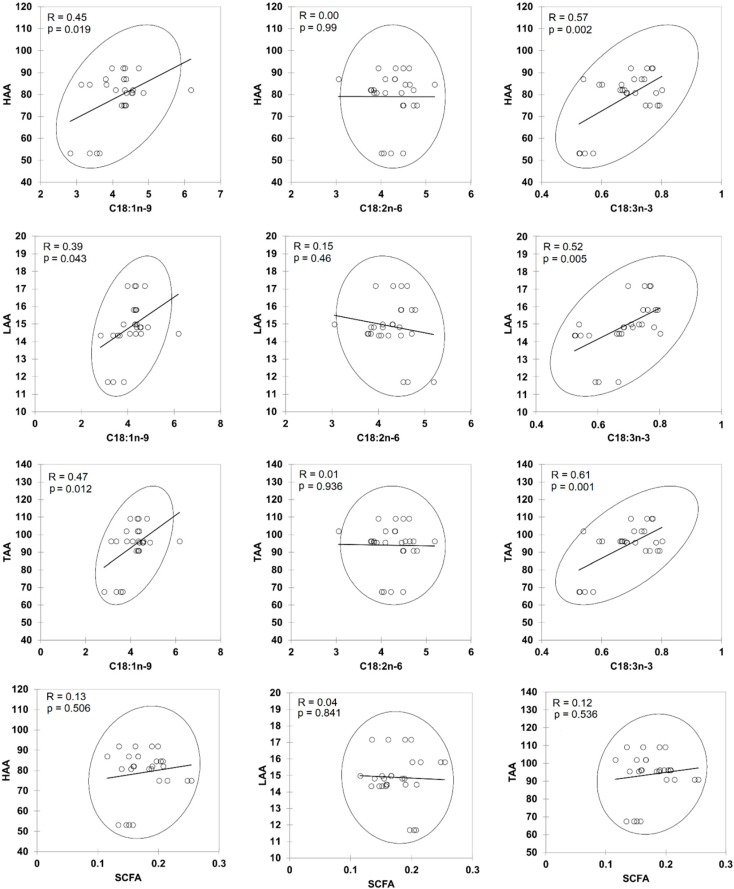
Scatter plots showing relationships between the antioxidants activities and unsaponified fatty acids in different commercial natural soaps after cold saponification. HAA = hydrophilic antioxidant activity, LAA = lipophilic antioxidant activity, TAA = total antioxidant activity. Short Chain Fatty Acids (SCFA) = C8:0–C10:0.

Considering we observed that the majority of unsaturated fatty acids present in the natural soap feedstocks were retained as unsaponified fatty acids in the soaps produced, we wanted to assess the antioxidants, phenolics, and oxidants levels in the soaps to determine if they would have any relationships with preserving the unsaturated unsaponified fatty acids in natural soaps. Cold saponification was used to manufacture the natural herbal soaps evaluated in this study (Figure 1). This method of saponification is preferred by artisanal soap makers because it offers more creative flexibilities with the choice of natural ingredients that can be used in the formulation, as well as potentially preserving the antioxidants, free fatty acids, essential oils, and plant extracts used as additives in the formulation [1,3]. In the present study, we evaluated the phenolic, antioxidant, and oxidant content of natural herbal soaps formulated with high-quality plant-based feedstock (vegetable oils) and additives (plant extracts and essential oils). We observed cold saponification resulted in the retention of hydrophilic (HPC), lipophilic (LPC), and the total phenolic content (TPC) in all the natural herbal soaps evaluated in this study (Figure 4a–c). Hydrophilic compounds (phenolic, antioxidants, or oxidants) are soluble in water or aqueous solvents. Lipophilic compounds, on the other hand, are soluble in organic solvents [9]. The highest TPC was observed in BB (15.88 ± 0.36), FGA (16.21 ± 0.94), BBR (20.40 ± 1.00) and FGR (13.46 ± 1.17 µmol quercetin equivalents/g soap). The plant oils (soap feedstock) and extracts used as additives to formulate the natural soap products are excellent sources of polyphenols [10], and as such, contributed to the hydrophilic, lipophilic, and total phenolic content remaining in the natural soaps after cold saponification. One interesting observation was that the H soap formulation poorly retained the phenolics, particularly the lipophilic phenolics following cold saponification (Figure 4). The use of additives (rosemary extract and essential oils), on the other hand, increased the TPC content in BB and FG natural soaps manufactured using cold saponification. Polyphenols are known to have potent antioxidant activities [9,10,11]. Additionally, the additives and plant oils used as ingredients to formulate the natural herbal soaps could also contain compounds, such as vitamin E, carotenoids, etc., with antioxidant activities [3,10]. The base bar formulated with rosemary extract (BBR) as an additive contained the highest total antioxidant activity (109.22 ± 5.55 µmol Trolox equivalents/g soap). However, the antioxidant activities were similar between the other natural soaps (BB, FG, H, and FGR), except in HA, where a significantly lower level was observed (67.64 ± 1.03 µmol Trolox equivalents/g soap). The hydrophilic antioxidants accounted for the largest component of the total antioxidant activities (Figure 4). Cold saponification is considered a milder form of soap saponification [1] and is preferred by some artisanal soap makers because it can be effective in retaining the fragrance and antioxidants present in the soap ingredients used to formulate natural soaps [3], consistent with the findings observed in this study. We also observed that 65% of the hydrophilic antioxidant activity (HAA) came from the HPC, and 72% of both the lipophilic (LAA) and total (TAA) antioxidant activity came from the LPC and TPC present in the natural herbal soaps (Figure 5). This is the first time to our knowledge that this relationship is reported in natural herbal soaps manufactured using cold saponification. Considering that we observed between 60% and 100% of the unsaturated fatty acids existing in the plant oils used as soap feedstock were retained as unsaponified fatty acids in the final products (Figure 3), we assessed the oxidation status of the natural soaps following cold saponification and curing since unsaturated fatty acids are known to be susceptible to lipid oxidation [3,11,12]. The results indicated that significant oxidation existed in the natural soaps evaluated in this study, with the hydrophilic oxidants being the major contributor to the total oxidant status (Figure 4). The lowest oxidation status was observed in BB (27.06 ± 1.17) and FGA (30.40 ± 2.31 µmol H_2_O_2_ equivalents/g soap). A significantly higher oxidation status was observed in FG (35.30 ± 1.34), H (37.45 ± 2.66), HA (35.49 ± 1.35), BBR (36.35 ± 1.01), and FGR (36.06 ± 1.01 µmol H_2_O_2_ equivalents/g soap). As such, the total (TOS) and lipophilic (LOS) oxidation status differentiated FG from H and BB, while the hydrophilic oxidation status (HOS) differentiated H from BB and FG. Polyphenols conferred 65–72% of the antioxidant activities observed in the natural soaps (Figure 5). One of the major functions of antioxidants is to suppress oxidation [9,11]. Polyunsaturated fatty acids present in the natural soaps as unsaponified fatty acids are highly susceptible to lipid oxidation [3,12]. Consequently, we determined if the observed soap polyphenols or antioxidants were associated with suppressed oxidation, thus enhancing the retention of unsaturated unsaponified fatty acids in the natural soaps following cold saponification. A high correlation was observed between the retention of C18:3n3 and TPC (r = 0.76), with the LPC (r = 0.78) being the major contributor to this overall relationship (Figure 6). This was concomitant with a significant correlation between the TAA and the retention of unsaponified C18:3n3 (r = 0.61) in the natural soaps following manufacturing by cold saponification (Figure 7). Similarly, the unsaponified C18:1n9 were significantly correlated with both the TPC (r = 0.57) and TAA (r = 0.47), albeit a weaker correlation compared to that C18:3n3 and TPC or TAA (Figure 6 and Figure 7). Surprisingly, there was no association between the retention of unsaponified C18:2n6 and TPC (r = 0.06) or TAA (r = 0.01) in the natural soaps evaluated in this study, suggesting that the retention of C18:2n6 may be associated with other antioxidant compounds, such as vitamin E, known to be incorporated in natural oils (used as soap feedstock) to stabilize or preserve the oils from oxidation [11,12]. The same observation was noted for the association between the shorter chain (C8:0–C10:0) unsaponified saturated fatty acids and TAA. Overall, these findings suggest phenolics and antioxidants present in the natural ingredients used to formulate herbal soaps were effectively retained by cold saponification, and that both the antioxidants and phenolics present in the feedstocks or additives were associated with the retention of C18:1n9 and C18:3n3 unsaturated unsaponified fatty acids in natural herbal soaps. These findings are of major significance to artisanal production of natural herbal soaps because antioxidants, polyphenols, and unsaponified unsaturated fatty acids appear to be major influencers or determinants of soap quality, consumer perception, and preference of the final products. 

### 2.4. Relationship between Sensory Attributes and Unsaponified Fatty Acid Composition of Natural Herbal Soaps

**Figure 8:**
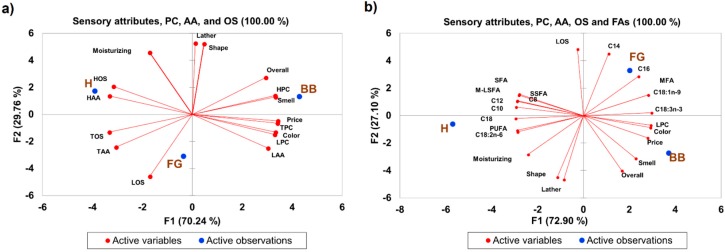
Principal component analysis showing the relationship between (**a**) sensory/perceptual attributes, phenolic content, oxidation status and antioxidant activity values; together with (**b**) the unsaponified fatty acid composition of commercial natural soaps manufactured using cold saponification. HOS = hydrophilic oxidant status, LOS = lipophilic oxidant status, TOS = total oxidant status, HPC = hydrophilic phenolic content, LPC = lipophilic phenolic content, TPC = total phenolic content, HAA = hydrophilic antioxidant activity, LAA = lipophilic antioxidant activity, TAA = total antioxidant activity. SFA = saturated fatty acids, PUFA = polyunsaturated fatty acids, SSFA = short chain saturated fatty acids (C8–C12), M-LSFA = medium/long chain saturated fatty acids (C14:0–C18:0).

Considering the levels and composition of unsaponified fatty acids were similar between the samples with and without additives (Table 2), we decided to focus our attention on determining the sensory perception of BB, FG, and H, as well as to determine if there was any relationship between sensory attributes and soap quality parameters based on the unsaponified fatty acid composition. Color and fragrance are two main sensory perceptual indicators of soap quality [3]. The BB had the highest color preference (7.37/10) and appealing smell (6.4/10) compared to FG (5.4/10 for color and 4.6/10 for appealing smell), and H (1.8/10 for color and 4.1/10 for appealing smell) (Table 3). This was very interesting because the base bar was formulated as part of the experimental design, where it was used as a control bar to compare the effects of soap formulation across samples. The other soaps were formulated with exotic essential oils as additives, but this did not translate to superior overall acceptance. Shape, lather, moisturizing ability, and estimated price were also evaluated in this study. However, there was no significant difference between the soap types and these sensory parameters. As such, the overall perception and preference were similar between all three soap types (Table 3). Pearson’s correlation was used to determine if any relationships existed between the natural soaps’ sensory attributes. The following sensory attributes were observed to be significantly correlated with each other (Table 4): vibrant color with appealing smell (r = 0.86) and price estimate (r = 0.98), appealing shape with lather (r = 0.99), appealing smell or fragrance with the estimated price (r = 0.93) and overall rating (r = 0.96), and lather with moisturizing abilities (r = 0.85). These relationships suggest that an appealing smell had the greatest influence on consumer preference, and that color and appealing smell influenced perceived pricing of the natural soaps evaluated in this study. Thus, it seems that fragrance (appealing smell) and color were the best indicators of natural soaps’ perceived quality. Pricing, smell (fragrance), and color are major affective response indicators reported in the literature. Affective response is used as an instrument to measure market potential [13]. Surprisingly, lather and moisturizing characteristics (major indices of natural soap quality) [1,2,3] had very little influence on the overall consumer ratings of the three natural soaps evaluated in this study. However, the overall preference or affective response was similar between the three natural soaps (Table 3). Consequently, we sought to determine whether the unsaponified fatty acids observed in the natural soaps had any influence on the sensory qualities or attributes of the soaps evaluated in this study. This is of relevance because the unsaponified fatty acids can modulate soap quality, performance, and consumers’ acceptance or preference [2,3]. Overall, when sensory attributes, antioxidant activities, phenolic content, and oxidation status were used to cluster or differentiate the three natural soaps, the soaps clustered in different quadrants of the biplot following PCA analysis (Figure 8). The BB was grouped in quadrant 1 based on the lather ability, appealing shape, overall rating, appealing smell, and the level of HPC. FG was grouped in quadrant 3 of the biplot based on TAA, LOS, and TOS, while H was grouped in quadrant 4 based on moisturizing abilities, HOS, and HAA. This grouping explained 100% of the variance present in the sample (Figure 8a). Similarly, when the unsaponified fatty acids were added to the model and PCA analysis conducted, H, FG, and BB segregated into separate quadrants of the biplot (Figure 8b). Unsaponified C14:0, C16:0, C18:1n9, and C18:3n3 differentiate FG, while H was differentiated by C18:0, C18:2n6, and overall PUFA content. BB was not segregated based on unsaponified fatty acids. Color, LPC, price, smell, and overall preferences were instead observed as the parameters segregating BB in quadrant 2 of the biplot. This grouping also explained 100% of the variance present in the sample (Figure 8b). Interestingly, BB consistently had the best overall preference of the soaps formulated in this study (Figure 8). These findings also indicate HPC (Figure 8a) and LPC (Figure 8b) appeared to be useful extrinsic cues that were perhaps associated with consumers’ perception of natural soap quality and could potentially predict affective response. 

Taken altogether, appealing smell appeared to have the greatest influence on consumers’ preference, and that color and appealing smell influenced the perceived pricing of natural soaps. The BB had superior color ratings and appealing smell compared to the other soaps following sensory evaluations. Overall, when unsaponified fatty acids, antioxidants, phenolics, oxidation status, and sensory attributes were used to group the natural soaps, BB had the best overall preference. This is a significant finding because the base bar was included in this study as the control and was not a part of the more sophisticated formulations using specialty plant oils (as feedstock), exotic essential oils, or plant extracts (as additives) that the industry partner typically used to make their natural soaps for commercial sales. The use of these ingredients did not translate to superior consumer preference or acceptance. These findings suggest some of the more exotic additives and specialty oils used as feedstock in manufacturing natural soaps may not be producing the perceived consumer acceptance or preference, and considerations should be given to their use during manufacturing of hand-made natural soaps. This could have implications in the cost of materials used for production and potential profitability.

## 3. Material and Methods

### 3.1. Soap Oils 

Three natural herbal soaps designated base bar (BB), forest grove (FG), and hibiscus rose hip (H) were manufactured using cold saponification. The health Canada compliant numbers for each of the natural soaps are as follows: 006324, 0.006354, and 0.006362 respectively. The base bar was designated the control bar, and contained the following oils and butter melted and mixed together (*w*/*v*): coconut oil (30%), palm oil (20%), soybean oil (20%), olive oil (20%), castor oil (5%), and shea butter (5%). The forest grove soap was manufactured from the base bar ingredients with the following modification: soybean oil was reduced to 18%, and 2% stearic acid was added to compensate for the reduced soybean oil. The hibiscus rose hip soap was also manufactured using the base bar ingredients with the following modification: soybean and olive oils were reduced to 18% and 10%, respectively, and 2% stearic acid and 10% rice bran oils were added instead to compensate for the reduced levels of soybean and olive oils. The oils/butter mixture for each soap type were melted in a double jacketed melter at 150 °F and thoroughly mixed using an industrial immersion blender. Aliquots (8 g) of the melted oils and butter from each soap batch were obtained to determine the chemical composition of the soap feedstock prior to cold saponification. The oil samples (soap feedstock) were designated OilBB (oil from base bar), OilFG (oil from forest grove), and OilH (oil from hibiscus rose hip). Four separate batch (replicates) were independently made for each soap type.

### 3.2. Cold Saponification Soap Production

Each batch of soap was manufactured based on a proprietary commercial recipe developed by the industrial collaborator using 35% water and 6% superfat (lye discount). The saponification value for all oils used in the composition of the different soaps were determined using a commercial lye calculator. The cold process method herein referred to as cold saponification was used for soap production where lye (pure sodium hydroxide) was used as the base in the saponification process. The amount of sodium hydroxide required (determined from the lye calculator) was mixed with water and left to cool for 60 min. The cooled lye and water was added to the melted oils and butter for each soap type (see above), and the subsequent mixture was blended until thickened (about the consistency of thick cream), but before trace (point where the saponification process is almost complete) was reached (the consistency of pudding). The mixture was used to fill parchment paper-lined molds, and the soaps were left to complete saponification at room temperature for 24 h. Following saponification and removal from the mold, each loaf of soap weighed approximately 1500 g, and measured 2 × 3 × 15 inches. A commercial wire loaf cutter was used to cut the loaves into 15 identical bars simultaneously, with each bar having the same shape and dimensions (measured approximately 1 × 2 × 3 inches, and weighed 100 g), and the soaps left to cure for 4 weeks. After curing, 8 g were cut with a scalpel blade from the center of each soap bar, wrapped in aluminum foil, and stored at −80 °C for chemical analyses. A total of eight batches for each soap type were made for both chemical and sensory perception analyses. Each soap type was designated as follows: base bar (BB), forest grove (FG), and hibiscus rose hip (H) based on their formulation.

### 3.3. Addition of Natural Additives to Herbal Soaps

To evaluate the effects of additives on the unsaponified fatty acid composition of BB, FG, and H herbal soaps following cold saponification, natural plant additives were added to each of the soaps as follows: To the base bar and forest grove soaps, 0.05% (*w*/*w*) of rosemary extract (Saffire Blue, Toronto, ON, Canada) (containing organic coconut oil, rosemary, natural vitamin E, and a proprietary blend of non-allergic plant extracts) was added to each loaf. Both soap samples were designated BBR (base bar + rosemary extract) and FGR (forest grove + rosemary extract), respectively. To both hibiscus and forest grove, a second combination of natural additives consisting of a combination of naturally sourced colored clays (for color) and essential oils (for fragrance) were added. Specifically, 0.05% (*w*/*w*) of French green clay and essential oil blend containing patchouli and fir needles, cedar leaf, cedar wood, and cinnamon bark were added to the forest grove bar. This soap bar was designated FGA. To hibiscus, French pink clay, filtered hibiscus rose hip tea steeped in boiled water for 1–3 h (for color, aesthetic appeal, and fragrance), and an essential old blend containing sweet orange, pink grape fruit, bergamot, vanilla, cinnamon bark, cedar leaf, ylang ylang, cananga, lavender, and palmarosa were added as additives. This soap bar was designated HA. The amount of additives added to each soap type was determined based on the FDA approved usage of extracts/natural preservatives in cosmetics (0.02–0.05% of finished product weight). As such, 0.05% (750 mg) of the finished product weight (1500 g) was used to add the additives in this study. The additives were added to the mixture and thoroughly mixed just before molding. The same cold saponification method noted above was used to manufacture the soaps with additives. Eight grams was cut with a scalpel blade from the center of each soap bar, wrapped in aluminum foil, and stored at −80 °C for chemical analyses.

### 3.4. Chemical Analysis of the Natural Herbal Soaps

#### 3.4.1. Sample Extraction

Extraction was carried out according to the methods of Thomas et al. [9] and Cano et al. [14] with slight modifications in order to obtain the hydrophilic and lipophilic phenolic content, antioxidant activities, and oxidation status of each natural soap. Samples from each batch of soap (100 mg) were weighed (4× replication) in glass centrifuge vials. The soaps were cut into small pieces and 1 mL of HPLC grade acetone: ethanol (1:1 *v*/*v*) were added. The samples were centrifuged at 10,000× *g* for 15 min, and the supernatant was carefully removed without disturbing the pellet. The supernatant was filtered with glass wool, and the filtrate was used without further dilution to determine the lipophilic antioxidant activity and the organic phenolic content of the soaps. The undisturbed pellets were re-suspended in 1 mL of cold 50 mM sodium phosphate buffer (pH 7.5), and centrifuged at 10,000× *g* for 15 min, and the supernatant was carefully removed without disturbing the pellet. The supernatant was diluted 1:10 with 50 mM sodium phosphate buffer. This diluted supernatant was used to determine the hydrophilic antioxidant activity and aqueous phenolic content.

#### 3.4.2. Total Phenolic Content (TPC) Analysis

The hydrophilic and lipophilic phenolic content were determined separately using a 10-fold diluted solution of Folin–Ciocalteu reagent with quercetin as a standard in the range of 0–1.0 mg/mL [9,14]. The aqueous phenolics (soluble in aqueous solution) and organic phenolics (soluble in organic solvent) were determined by adding 5 µL of the samples extracted with the buffer or organic solvent, respectively. To the sample mixture, 130 µL of Folin–Ciocalteu reagent and 75 µL of either buffer or ethanol:acetone (1:1 *v*/*v*) extract was added to microplate wells to determine the hydrophilic and lipophilic phenolic content, respectively. The microplates with the mixture were incubated in the dark at room temperature for 30 min, and the absorbance was measured at 755 nm using a Synergy HT microplate reader (Biotek, Fisher Scientific, Mississauga, ON, Canada). The results were expressed as micromole quercetin equivalents per gram of soap. Four replicates were analyzed per standard concentration or sample treatment. Values for total phenolic content were determined by summation of the aqueous (hydrophilic) and organic (lipophilic) phenolic values.

#### 3.4.3. Ferric Reduction Antioxidant Power (FRAP) Method

The hydrophilic and lipophilic antioxidant activities were determined according to the methods of Jimenez-Alvarez et al. [15]. This method is based on the capacity of a sample to scavenge the FRAP radical cation compared to a standard antioxidant (Trolox) in a dose–response curve (0–100 µM). The working solution (reaction mix) was prepared fresh daily by adding 25 mL of 30 mM acetate buffer (pH 3.6), 2.5 mL of 10 mM tripyridyltriazine (TPTZ) solution, and 2.5 mL of 20 mM FeCl_3_·6H_2_O solution. This solution was heated to 37 °C in an oven for 15 min before use. A standard solution of 1 mM of Trolox was made using 6.25 mg Trolox in 25 mL of sodium phosphate buffer for the hydrophilic antioxidant activity or acetone:ethanol (1:1 *v*/*v*) for the lipophilic antioxidant activity. For hydrophilic and lipophilic antioxidant activities, 10 µL of sample or standard was added to the reaction medium to give a total volume of 200 µL in each microplate well. The sample mixture was incubated in the dark for 30 min and the absorbance was measured at 593 nm using a Synergy HT microplate reader (Biotek, Fisher Scientific, Mississauga, ON, Canada). Four replicates were analyzed per standard concentration or sample treatment. The results were expressed as micromole Trolox equivalents per gram soap. Values for total antioxidant activity were determined by summation of the aqueous (hydrophilic) and organic (lipophilic) antioxidant values.

#### 3.4.4. Total Oxidant Status Analysis

This analysis was conducted using the established method of Erel [16]. The principle of this method is that oxidants present in the soap samples will oxidize ferrous ions and an *o*-dianisidine complex in the assay to produce ferric ions. The ferric ions produce a chromophore (colored complex) with xylenol orange in an acidic medium, and the amounts of oxidant molecules present in the samples can be determined via a linear relationship with known standards by measuring the absorbance at 560 nm. Briefly, a stock solution of Reagent 1 was prepared by dissolving 114 mg of xylenol orange and 8.18 g of sodium chloride in 900 mL of 25 mM H_2_SO_4_ (1.2 mL of 98% H_2_SO_4_ (d = 1.84 g/L) in 900 mL H_2_O). This was followed by the addition of 100 mL of glycerol to give a final composition for Reagent 1 of 150 µM xylenol orange, 140 mM NaCl, 1.35 M glycerol, and a pH of 1.75. Reagent 2 was prepared by dissolving 1.96 g of ferrous ammonium sulfate and 3.17 g of *o*-dianisidine dihydrochloride in 1000 mL of 25 mM H_2_SO_4_ (1.33 mL of 98% H_2_SO_4_ (d = 1.84 g/L) in 1000 mL of H_2_O). This reagent had a composition of 5 mM ferrous ammonium sulfate and 10 mM *o*-dianisidine dihydrochloride. Hydrogen peroxide in water was used as internal standards, where 10.24 µL of 30% H_2_O_2_ was added to 100 mL of deionized water to give a 1 mM stock solution. From this stock solution, serial dilutions were performed to give 0, 25, 50, 100, and 150 µM. For this assay, 35 µL of sample or standard was placed in microplate wells along with 225 µL of Reagent 1 and 10.6 µL of Reagent 2. The absorbance was then read at 560 nm using a Biotek Synergy HT microplate reader (Fisher Scientific, Mississauga, ON, Canada). Four replicates were analyzed per standard concentration or sample treatment, and the lipophilic and hydrophilic oxidant status was determined. The results were expressed as µM equivalents of H_2_O_2_ per gram of soap. Values for the total oxidant status were determined by summation of the aqueous (hydrophilic) and organic (lipophilic) oxidant values.

#### 3.4.5. Soap Lipid Extraction

Soap lipids were extracted using the established Bligh and Dyer method with the following modifications [17,18]. Briefly, 50 mg of sample was weighed into glass centrifuges tubes in four replicates, and 1 mL of methanol containing 0.01% butylated hydroxytoluene (BHT) was added. Nonadecanoic acid (C19:0) was added as an internal standard to each centrifuge tube (100 µL of 0.2 mg/mL). The sample mixture was thoroughly vortexed, then 1 mL chloroform and 0.8 mL distilled water were added, and the mixture was centrifuged at 10,000× *g* for 10 min. The organic layer was retained to determine the unsaponified fatty acids in the soaps or fatty acids in the oils. The fatty acids in the mixture were methylated by taking 200 µL aliquots of the organic layer from each sample, then dried under nitrogen gas. To the dried residue, 200 µL of methanolic-HCl (3N) was added, and the samples were vigorously vortexed then incubated overnight at room temperature. Following incubation, 800 µL of deionized water was added and the samples were extracted three times using 500 µL of n-hexane each time. The hexane fractions from each sample were pooled (1500 µL), 100 µL of water scavenger (DMP) was added, and the sample was dried under nitrogen [19]. The methylated fatty acids were then re-suspended in 100 µL of n-hexane, vigorously vortexed, transferred to insert in GC vials, and then analyzed via gas chromatography-mass spectrometry/flame ionization detection (GC-MS/FID). 

#### 3.4.6. GC-MS/FID Analysis of FAMEs

GC-MS/FID analysis was conducted on a Thermo Scientific Trace 1300 gas chromatography (GC) (Mississauga, ON, Canada) coupled to a Thermo Scientific TSQ 8000 Triple Quadrupole mass spectrometer (MS) (Burlington, ON, Canada) or a flame ionization detector (FID) (Burlington, ON, Canada). Methylated fatty acids were separated with a DB23 high resolution column (60 m × 0.25 mm × 0.2 µm; Agilent Technology, Mississauga, ON, Canada) using helium as the carrier gas at a flow rate of 1 mL/min. One microliter (1 µL) of each sample was applied to the injection system in split mode (20:1) using a Tri-plus auto-sampler (Thermo Scientific, Burlington, ON, Canada). The oven temperature was programed as follows: the initial oven temperature of 80 °C was held for 0.5 min, then programmed to increase at 4 °C/min to 220 °C, held for 5 min at 220 °C, then increased at 4 °C/min to 240 °C, where it was held for 10 min. The methylated fatty acids were determined from the comparison of retention times and mass spectra were obtained from commercial standards (Supelco 37 component mix, Supelco PUFA No. 3, Supelco FAME mix C8-C24, C16 DMA, and C10 DMA; Sigma Aldrich, Oakville, ON, Canada) and the NIST database (Thermo Scientific, Burlington, ON, Canada). The amounts of individual fatty acids identified were calculated using standard curves prepared from the standard mixtures, and values were presented as µg/mg soap [18].

### 3.5. Soap Sensory Analysis

The effects of the unsaponified fatty acids on the perception of herbal soaps were conducted according to the methods of Garcia-Segovia et al. [20] and Sampaio et al. [21] following ethics approval by the Grenfell Campus-Memorial University Research Ethics Board. Fifty-nine untrained participants were recruited from the student, staff, and faculty at Grenfell Campus of Memorial University who had no prior knowledge of the soap treatments. Soaps from each treatment (BB, FG, and H) were presented to participants in a random order. Participants were asked to complete a questionnaire following multiple uses of the soap, and ranked the soaps based on color, appealing shape, fragrance, lather, moisturizing effects, estimated price (were asked to give a price for each product), and overall preference. Acceptance and preference for each product was scored on a structured hedonic scale [20,21] assigned using the sensory analysis software (SIMS 2000, version 6.0., Berkely Heights, NJ, USA). The ratings obtained from the participants were correlated with the outputs from the chemical analyses (unsaponified fatty acid composition, antioxidant activities, phenolic contents, and oxidation status) to determine relationships between the unsaponified fatty acid composition and sensory perception of the natural herbal soaps. 

### 3.6. Statistical Analysis

All measurements of chemical parameters (fatty acids, antioxidant activities, phenolic contents, and oxidation status) were made in quadruplet. Analysis of variance (ANOVA) was used to determine the effects of treatments on chemical parameters. Where treatment effects were significant, the means were compared using Fisher’s LSD test with α = 0.05. For sensory perception analysis, results from the hedonic scale were analyzed using ANOVA and frequency distribution analysis, and means (*n* = 59) were separated using Fisher’s LSD test (α = 0.05) when significant differences were observed [21]. To test the linear relationships between chemical characteristics and sensory perception output, Pearson’s correlation coefficient was used. Principal component analysis was used to discern relationships between sensory perception and chemical parameters for each soap type, and to assess the influence of the unsaponified fatty acid composition on sensory perception of the tested commercial natural herbal soaps. The sensory perception analysis was done using the sensory information management system software (SIMS 2000, version 6.0, Berkely Heights, NJ, USA). All other data analysis was done using XLSTATS (Addinsoft, Paris, France).

## 4. Conclusions

There is currently a paucity of information in the literature related to how the content of unsaponified fatty acids in natural herbal soaps manufactured using cold saponification influences the sensory perception and consumer’s preference or acceptance of natural soap products. The output from this study attempted to fill this knowledge gap. We observed commercial natural soaps manufactured from plant oils and additives as feedstock by cold saponification retained significant levels of unsaponified fatty acids, phenolic compounds, and antioxidant activities in the final products. The majority of the unsaturated fatty acids present in the feedstock remained unsaponified in the natural soaps after manufacturing by cold saponification. The unsaponified monounsaturated fatty acids (C18:1n9) appeared to be the most responsive to the variation in the choice of vegetable oils used as soap feedstock during manufacturing via cold saponification. Addition of additives in the soap formulation had a significant effect on the percent change or retention of the feedstock fatty acids as unsaponified fatty acids in the finished products. Both the antioxidants and phenolics were associated with the retention or levels of C18:1n9 and C18:3n3, but not C18:2n6 unsaturated unsaponified fatty acids in natural herbal soaps manufactured via cold saponification. These findings suggest cold saponification is an effective approach to enhance the levels of unsaponified fatty acids, phenolics, and antioxidants in hand-made natural soaps. Appealing smell was the greatest influencer of consumer preference, while color and appealing smell were the best indicators of natural soaps’ estimated pricing and consumers’ perceived quality, and consequently acceptability. These findings are of major significance to artisanal production of natural herbal soaps because antioxidants, polyphenols, and unsaponified unsaturated fatty acids appear to be major determinants of natural soap quality, consumer perception, and preference of the final products. One of the most significant findings in this study was that the base bar used as control in this study had the best overall preference compared to the other soaps formulated with specialty or exotic plant oils as feedstock and additives. These findings suggest some of the more exotic additives and specialty oils used to manufacture hand-made natural soaps may not be producing the perceived consumer acceptance or preference, and that artisan natural soap makers should give careful considerations to their use during the manufacturing of hand-made natural soaps. This work provides some baseline information regarding natural hand-made soaps manufacturing, which is very sparse in the scientific literature. The hope is that the information presented will stimulate additional studies by other researchers in the scientific community, to further improve the knowledge that may be of value to the growing specialty hand-made soap industry. 

## Figures and Tables

**Table 1:** Fatty acid composition of the combined oils and butter used to manufacture different natural soaps.

Fatty Acids	Oil BB	Oil FG	Oil H
C8	0.99 ± 0.16 ^a^	1.32 ± 0.44 ^a^	2.49 ± 0.19 ^b^
C10	0.72 ± 0.11 ^a^	0.91 ± 0.31 ^a^	1.72 ± 0.15 ^b^
C12	4.92 ± 0.43 ^a^	6.25 ± 1.95 ^a^	11.67 ± 1.48 ^b^
C14	1.79 ± 0.13 ^a^	2.09 ± 0.65 ^a^	3.65 ± 0.43 ^b^
C16	16.61 ± 3.79 ^a^	23.94 ± 7.19 ^a^	47.05 ± 5.72 ^b^
C18	3.13 ± 1.26 ^a^	3.55 ± 1.72 ^a^	3.78 ± 0.56 ^a^
C18:1n-9	3.93 ± 0.56 ^a^	4.66 ± 1.63 ^a^	5.32 ± 0.88 ^a^
C18:2n-6	4.60 ± 0.31 ^a^	4.77 ± 1.85 ^ab^	6.71 ± 0.21 ^b^
C18:3n-3	0.76 ± 0.06 ^a^	0.75 ± 0.30 ^a^	0.73 ± 0.02 ^a^
SFA	28.17 ± 5.84 ^a^	38.05 ± 12.21 ^a^	70.36 ± 7.48 ^b^
MUFA	3.93 ± 0.56 ^a^	4.66 ± 1.63 ^a^	5.32 ± 0.88 ^a^
PUFA	5.36 ± 0.37 ^a^	4.62 ± 1.42 ^a^	7.44 ± 0.23 ^b^

Values (µg/mL) represent means ± standard errors. Means in the same row accompanied by different letters are significantly different between treatments at Fisher’s LSD = 0.05, *n* = 4 per experimental replicate. SFA = sum of the saturated fatty acids (C8 to C18), MUFA = sum of the monounsaturated fatty acids (C18:1n9), PUFA = sum of the polyunsaturated fatty acids (C18:2n6 + C18:3n3). The lipid components in the table are arranged based on the fatty acid composition with the number before the colon representing total number of carbons, while the numbers after the colon represents the total number and position of first double bonds (e.g., C16:3n3 = 16 carbons with 3 double bonds; first double located at carbon 3 counting from the terminal end). Natural soap acronyms: BB = base bar (control), FG = forest grove, H = Hibiscus rose hip.

**Table 2:** The effects of cold saponification and natural additives on the unsaponified fatty acid composition in different commercial natural soaps.

Soaps without Additives				Soaps + Natural Additives			
Fatty Acids	BB	FG	H	FGA	HA	BBR	FGR
C8	0.08 ± 0.01 ^ab^	0.08 ± 0.01 ^ab^	0.10 ± 0.00 ^a^	0.11 ± 0.01 ^b^	0.06 ± 0.01 ^a^	0.08 ± 0.01 ^ab^	0.07 ± 0.01 ^a^
C10	0.09 ± 0.01 ^ab^	0.10 ± 0.01 ^ab^	0.11 ± 0.00 ^a^	0.12 ± 0.01 ^b^	0.08 ± 0.01 ^a^	0.09 ± 0.01 ^ab^	0.08 ± 0.01 ^a^
C12	1.06 ± 0.13 ^ab^	1.12 ± 0.15 ^ab^	1.21 ± 0.02 ^a^	1.39 ± 0.11 ^b^	0.98 ± 0.06 ^a^	1.11 ± 0.15 ^ab^	1.00 ± 0.14 ^a^
C14	0.56 ± 0.06 ^ab^	0.58 ± 0.06 ^ab^	0.56 ± 0.02 ^a^	0.70 ± 0.04 ^b^	0.48 ± 0.05 ^a^	0.59 ± 0.07 ^ab^	0.53 ± 0.07 ^ab^
C16	5.57 ± 0.48 ^ab^	5.85 ± 0.50 ^b^	5.21 ± 0.60 ^a^	5.96 ± 0.81 ^b^	5.03 ± 0.82 ^ab^	5.67 ± 0.52 ^b^	5.36 ± 0.49 ^ab^
C18	1.55 ± 0.32 ^a^	1.73 ± 0.68 ^a^	2.91 ± 0.12 ^ab^	2.83 ± 0.24 ^b^	2.38 ± 0.37 ^ab^	2.19 ± 0.32 ^ab^	1.88 ± 0.52 ^ab^
C18:1n-9	4.57 ± 0.20 ^c^	4.79 ± 0.95 ^c^	3.43 ± 0.36 ^a^	4.32 ± 0.05 ^bc^	3.34 ± 0.37 ^ab^	4.33 ± 0.31 ^bc^	4.20 ± 0.26 ^bc^
C18:2n-6	4.07 ± 0.28 ^a^	4.02 ± 0.46 ^a^	4.78 ± 0.35 ^a^	4.62 ± 0.16 ^a^	4.19 ± 0.22 ^a^	4.34 ± 0.30 ^a^	3.94 ± 0.60 ^a^
C18:3n-3	0.71 ± 0.05 ^bc^	0.70 ± 0.07 ^bc^	0.62 ± 0.04 ^a^	0.77 ± 0.02 ^c^	0.54 ± 0.02 ^ab^	0.75 ± 0.03 ^c^	0.68 ± 0.10 ^bc^
SFA	8.90 ± 0.96 ^ab^	9.46 ± 0.86 ^ab^	10.10 ± 0.73 ^a^	11.10 ± 1.09 ^b^	9.02 ± 0.89 ^ab^	9.73 ± 1.02 ^ab^	8.91 ± 1.21 ^ab^
MUFA	4.57 ± 0.20 ^c^	4.79 ± 0.95 ^c^	3.43 ± 0.36 ^a^	4.32 ± 0.05 ^bc^	3.34 ± 0.37 ^ab^	4.33 ± 0.31 ^bc^	4.20 ± 0.26 ^bc^
PUFA	4.78 ± 0.32 ^a^	4.72 ± 0.53 ^a^	5.40 ± 0.39 ^a^	5.39 ± 0.18 ^a^	4.73 ± 2.72 ^a^	5.09 ± 0.33 ^a^	4.62 ± 0.69 ^a^

Values (µg/mg) represent means ± standard errors. Means in the same row accompanied by different letters are significantly different between treatments at LSD = 0.05, *n* = 4 per experimental replicate. SFA = sum of the saturated fatty acids (C8 to C18), MUFA = sum of the monounsaturated fatty acids (C18:1n9), PUFA = sum of the polyunsaturated fatty acids (C18:2n6 + C18:3n3). Natural soap acronyms: BB = base bar (control), FG = forest grove, H = Hibiscus rose hip. FGA = forest grove + essential oil and clay, HA= hibiscus + essential oil and clay, BBR = base bar + rosemary, FGR = Forest grove + rosemary, HR = hibiscus + rosemary.

**Table 3:** Sensory attributes of different commercial natural soaps manufactured using cold saponification.

Parameter	BB	FG	H
Color	7.37 ± 0.35 ^c^	5.42 ± 0.40 ^b^	1.78 ± 0.21 ^a^
Shape	5.34 ± 0.38 ^a^	4.52 ± 0.35 ^a^	5.28 ± 0.37 ^a^
Smell	6.41 ± 0.33 ^b^	4.55 ± 0.39 ^a^	4.12 ± 0.33 ^a^
Lather	4.63 ± 0.30 ^a^	3.81 ± 0.33 ^a^	4.67 ± 0.33 ^a^
Moisturizing	4.07 ± 0.25 ^a^	3.70 ± 0.32 ^a^	4.35 ± 0.32 ^a^
Price	8.2 ± 0.53 ^b^	7.57 ± 0.52 ^ab^	6.30 ± 0.43 ^b^
Overall	5.29 ± 0.31 ^a^	4.68 ± 0.3 ^a^	4.74 ± 0.28 ^a^

Values represent means ± standard errors. Means in the same row accompanied by different letters are significantly different between treatments at LSD = 0.05, *n* = 59 per experimental replicate.

**Table 4:** Pearson’s correlation matrix demonstrating the relationship between sensory attributes of different natural soaps manufactured using cold saponification.

Attributes	Color	Shape	Smell	Lather	MoisturiZing	Price	Overall
Color	1	−0.113	**0.869**	−0.213	−0.696	**0.987**	0.703
Shape	−0.113	1	0.393	**0.995**	0.792	0.048	0.628
Smell	**0.869**	0.393	1	0.298	−0.250	**0.937**	**0.963**
Lather	−0.213	**0.995**	0.298	1	**0.850**	−0.054	0.546
Moisturizing	−0.696	0.792	−0.250	**0.850**	1	−0.572	0.022
Price	**0.987**	0.048	**0.937**	−0.054	−0.572	1	**0.807**
Overall	0.703	0.628	**0.963**	0.546	0.022	0.807	1

Bold values are significantly different at alpha = 0.05; *n* = 59 per experimental replicate.
